# Establishing gene models from the *Pinus pinaster* genome using gene capture and BAC sequencing

**DOI:** 10.1186/s12864-016-2490-z

**Published:** 2016-02-27

**Authors:** Pedro Seoane-Zonjic, Rafael A. Cañas, Rocío Bautista, Josefa Gómez-Maldonado, Isabel Arrillaga, Noé Fernández-Pozo, M. Gonzalo Claros, Francisco M. Cánovas, Concepción Ávila

**Affiliations:** Departamento de Biología Molecular y Bioquímica, Facultad de Ciencias, Universidad de Málaga, Campus de Teatinos s/n, E-29071 Málaga, Spain; Departamento de Biología Vegetal, Facultad de Farmacia, ERI Biotecmed, Universidad de Valencia, Avda. Vicent Andrés Estellés s/n, 46100 Burjassot, Valencia Spain; Boyce Thompson Institute for Plant Research, Cornell University, Ithaca, NY 14853 USA

**Keywords:** BAC, Bioinformatic pipeline, Gene capture, Gene model construct, Gene structure, Maritime pine, Promoter studies

## Abstract

**Background:**

In the era of DNA throughput sequencing, assembling and understanding gymnosperm mega-genomes remains a challenge. Although drafts of three conifer genomes have recently been published, this number is too low to understand the full complexity of conifer genomes. Using techniques focused on specific genes, gene models can be established that can aid in the assembly of gene-rich regions, and this information can be used to compare genomes and understand functional evolution.

**Results:**

In this study, gene capture technology combined with BAC isolation and sequencing was used as an experimental approach to establish *de novo* gene structures without a reference genome. Probes were designed for 866 maritime pine transcripts to sequence genes captured from genomic DNA. The gene models were constructed using GeneAssembler, a new bioinformatic pipeline, which reconstructed over 82 % of the gene structures, and a high proportion (85 %) of the captured gene models contained sequences from the promoter regulatory region. In a parallel experiment, the *P. pinaster* BAC library was screened to isolate clones containing genes whose cDNA sequence were already available. BAC clones containing the asparagine synthetase, sucrose synthase and xyloglucan endotransglycosylase gene sequences were isolated and used in this study. The gene models derived from the gene capture approach were compared with the genomic sequences derived from the BAC clones. This combined approach is a particularly efficient way to capture the genomic structures of gene families with a small number of members*.*

**Conclusions:**

The experimental approach used in this study is a valuable combined technique to study genomic gene structures in species for which a reference genome is unavailable. It can be used to establish exon/intron boundaries in unknown gene structures, to reconstruct incomplete genes and to obtain promoter sequences that can be used for transcriptional studies. A bioinformatics algorithm (GeneAssembler) is also provided as a Ruby gem for this class of analyses.

**Electronic supplementary material:**

The online version of this article (doi:10.1186/s12864-016-2490-z) contains supplementary material, which is available to authorized users.

## Background

Forests ecosystems play a fundamental role in the regulation of terrestrial carbon sinks and represent nearly 80 % of the world´s total plant biomass [1]. Conifers dominate a large part of the forests in the northern hemisphere, and they are intensively exploited as the primary source of wood for industrial purposes [2]. Conifers also exhibit unique characteristics among vascular plants, including: high genetic variability, long half-lives, seasonal survival, adaptation to secondary growth, and wood deposition among others [3]. Despite their economic and ecological importance, genomic studies of conifers have been hampered by the large size of their genomes, which range from 20 to 40 Gb, approximately 200 times the size of the *Arabidopsis* genome and approximately seven times the size of human genome [4]. However, recent technical advances in genomic sequencing have enabled the assembly of the Norway spruce [5], white spruce [6] and loblolly pine [7] genomes, and the sequencing of a number of additional species is underway [4, 8]. Although these assemblies represent landmark in conifer genomics, technological challenges continue to face the assembly and annotation of conifer genomes; they are characterized by a proliferation of retrotransposons, highly diverged repetitive sequences, accumulation of non-coding regions and extensive gene duplication [4, 8]. Also, large families of transposons and retrotransposons have been reported to occupy long stretches of the sequences in *Pinus* genomes [8, 9].

The analysis of BAC clones has been the most common approach used for genome characterization and in hierarchical sequencing projects, such as the human genome [10] or other genomes without available references [11]. The screening of BAC libraries has been used to target gene-rich regions in white spruce, but the approach has proven to be very laborious because most clones contain the non-coding regions of the genes, which is expected due to the large size of conifer genomes [12].

An alternative to obtaining the gene sequences of large and complex genomes is to perform an enrichment step to isolate the genomic DNA sequences of interest that contain the coding regions of genes by massive parallel sequencing and use them for further analysis [13]. This system named “gene capture”, uses rapid selective hybridization technique to obtain sequences of interest much more efficiently [14]. “-Gene capture-” has been widely used as a diagnostic tool for human whole exome analyses [15–17] but the use of the technique in plants has been much more limited [18, 19].

In this work, we used “-gene capture-” to elucidate the target, gene-rich regions in the genome of the maritime pine (*Pinus pinaster* L. Aiton), a conifer species of great ecological and economic importance in Europe and for which whole-transcriptome resources are available [20, 21]. To achieve this goal, 120-mer probes were designed from 866 tentative maritime pine transcripts, which include the probes for three characterized BAC clones as a control. These BAC clones were isolated by screening a maritime pine BAC library using specific cDNA probes [22] and then used as a reference for gene capture assays.

In this approach, megagametophyte calli haploid DNA from maritime pine was isolated, fractioned and bounded by a series of specific adapters for 454 sequencing. The captured genomic sequences were sequenced in an FLX-Titanium platform, and the reads were assembled and analyzed using the GeneAssembler bioinformatic pipeline to recover the gene models. This experimental approach also provided sequences for the proximal promoter region of the targeted genes. This can be used as initial information for genome walking to thoroughly characterize the *cis* elements contained in the regulatory region of these genes.

## Results

### BAC clone isolation and characterization

**Fig. 1:**
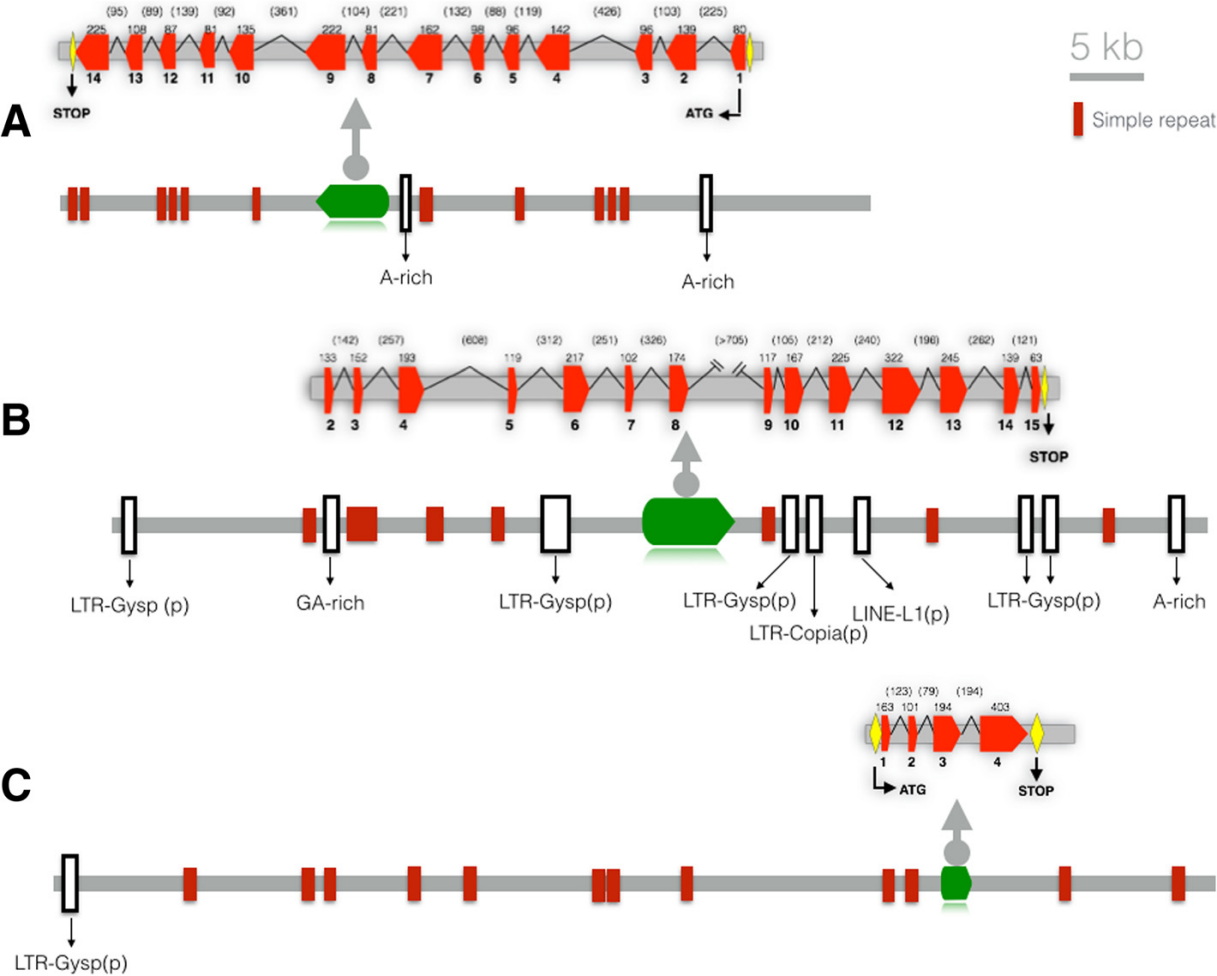
Structure of maritime pine genomic DNA contained in the BAC clones GenBank: KP172187 (**a**), GenBank: KP172184 (**b**) and GenBank: KP172195 (**c**) respectively. *Light red* boxes represent exons and introns the intervening line. The length in base pairs of each intron and exon is also indicated. Segments with similarity to transposable element and repetitive regions were identified with Repeat Masker and are represented by *white* and *dark red* boxes. The scale bar represents 5 kbp.

A *Pinus pinaster* BAC library that had been previously established in pools [22] was used to screen for particular clones containing gene coding sequences for asparagine synthetase 1 (*AS1*) [23] cDNA [GenBank: HQ625490], sucrose synthase (*SuSy*) cDNA [GenBank: AJ309093] and xyloglucan endotransglycosylase (*XET*) cDNA [GenBank: FN824804]. A detailed protocol for the identification and isolation of these BAC clones is described in the Methods section and summarized in the Additional file 1: Figure S1. As described in the Methods section, DNA was prepared from the purified BAC clones and fully sequenced, and the sequence assemblies for *AS1, SuSy* and *XET* were deposited in GenBank [GenBank: KP172187, GenBank: KP172194 and GenBank: KP172185 respectively]. Figure 1 depicts the corresponding BAC clone structures as single scaffolds. The sequences of the BAC clones were annotated and used to visualize the gene structure using GENote v.β. [23], which was used to detect the presence of the gene, its promoter location, the putative intron-exon pattern and the presence of transposable elements. The *AS1* sequence in the BAC clone exactly matches the previously characterized maritime pine *AS1* cDNA [24], and Fig. 1a shows the pattern of the BAC clone containing the *AS1* gene assembled in a single scaffold of 46,111 bp. The *AS1* gene is organized into 14 exons spanning a region of 3974 nucleotides, and the processed length without introns corresponds to a gene product with 1782 nucleotides that yields a protein with 593 amino acids. By comparing the *Arabidopsis* and poplar *AS* sequences in the databases, we determined the exon and intron structures, which are presented in Tables 1 and 2, respectively. The size and number of exons contained in the BAC clone are closer to those of the *ASN3* (At5g10240) gene from *Arabidopsis*. A total of 13 introns were identified, and their relative positions with respect to the coding region are well conserved compared to the *Arabidopsis ASN3* gene as well as the *P. trichocarpa* Potri.005G075700 model (Table 2).

**Table 1:** Exon length comparisons among complete *AS* genes from *P. pinaster* and *AS* from two angiosperm plants

Exon length (nt)	*Pinus pinaster AS1*/*AS3*/*AS5*	ASN_BAC	*Arabidopsis thaliana ASN3* (At5g10240)	*Arabidopsis thaliana ASN1* (At3g47340)	*Populus trichocarpa* AS (Potri.009G072900)	*Populus trichocarpa* AS (Potri.005G075700)
E1	80	80	80	80	80	80
E2	139	139	139	139	139	139
E3	96	96	100	96	96	96
E4	142	142	135	333	142	142
E5	96	96	96		191	96
E6	98	98	98			95
E7	162	162	162	162	162	162
E8	81	81	84	303	81	81
E9	222	222	222		222	222
E10	135	135	129	135	135	135
E11	81/84*	81	81	168	81	81
E12	87	87	87		87	87
E13	108	108	108	108	108	108
E14	252/243/240	252	78	231	246	231

*Exon from *AS5*. The gene capture model is also included

**Table 2:** Intron length comparisons among complete *AS* genes from *P. pinaster* and *AS* from two angiosperm plants

Intron length (nt)	*Pinus pinaster AS1*	*Pinus pinaster AS3*	*Pinus pinaster AS5*	ASN_BAC	*Arabidopsis thaliana ASN3* (At5g10240)	*Arabidopsis thaliana ASN1* (At3g47340)	*Populus trichocarpa* AS (Potri.009G072900)	*Populus trichocarpa* AS (Potri.005G075700)
I1	225	213	124	225	260	422	509	78
I2	101	88	112	101	186	88	156	227
I3	426	136	422*	426	192	82	152	369
I4	119	133	126	119	102	78	94	263
I5	88	112	117	88	184		115	520
I6	132	213	139	132	94			721
I7	219	261	221	219	85	136	89	533
I8	104	104	103	104	84	80	93	81
I9	87	87	87	87	83		96	136
I10	92	89	99	92	209	83	120	453
I11	139	100	109	139	85	91	85	110
I12	89	104	102	89	91		91	236
I13	95	95	90	95	96	93	100	152

*Incomplete Intron. The gene capture model is also included

A similar analysis of the structure of the *SuSy* and *XET* genes was performed. The *SuSy* BAC clone is represented in a single scaffold of 59,327 bp (Fig. 1b), and the gene sequence is organized into 14 exons encoding a protein with 769 amino acids. The comparison to the *SuSy* gene models in other plants indicated that the 5´end of the gene is missing in the pine BAC clone obtained for this study. The lengths and composition of the exon and intron are presented in Additional file 2: Table S1 and Additional file 3: Table S2, respectively, and a comparative study showed a high degree of conservation in the number and size of the exons in the maritime pine BAC model compared with the *SuSy2* gene from *Arabidopsis* and the POPTRDRAFT_830445 gene from *P. trichocarpa.* In contrast, substantial differences in the number and lengths of the exons were found when compared with *SuSy3* from *Arabidopsis* and POPTRDRAFT_826368 from *Populus*.

The *XET* BAC clone is represented in a single, 64,767-bp scaffold (Fig. 1c), and the sequence renders a model organized into 4 exons and 3 introns encoding a protein with 287 amino acids. The lengths and distribution of the exons and introns of the gene contained in the BAC clone are represented in Additional file 4: Table S3 and Additional file 5: Table S4, respectively. Two *Arabidopsis* gene models*, XET 9* (At4g032210) and *XET 3* (At3g25050), were closest to the pine gene sequence, but it was impossible to determine which is more similar to the gene contained in the pine BAC.

Considering that conifer genomes are characterized by retrotransposon proliferation and extensive regions containing repetitive elements, we performed a deeper study of the presence of these elements in the BAC clones. Through a comparison with the corresponding BAC-containing-gene available in *Picea glauca* (no BAC corresponding to *XET* was available), we analyzed the presence of repetitive elements. The content and type of the repetitions in both conifer BACs are summarized in Table 3. The percentage of bases that are part of the retroelements is quite similar in both *SuSy* BACs. But this percentage is significantly lower in the *AS* pine BAC, probably due to the different lengths of both BAC clones. In terms of the types of repeated elements, the retroelements, LTRs and Gypsy/Dir1 are the most represented in both *SuSy* BACs as well as in the *P. glauca AS* BAC with similar percentages of the total number of bases. DNA transposons, Simple repeats and low complexity elements contribute less to the size of the BAC.

**Table 3:** Content and type of repeats present in *SuSy* and *AS1* BACs from *P. glauca* and *P. pinaster*

	*Susy P. glauca*	*Susy P. pinaster*	AS *P. glauca*	AS *P. pinaster*
GeneBank:	KC860252	KP172192	KC860234	KP172187
Total length:	137047 bp	59397 bp	130154 bp	46111 bp
GC level:	38.66 %	39.03 %	38.17 %	34.80 %
	Number of elements/percentage of sequence			
Bases masked:	10259 bp (7.49 %)	5173 bp (8.71 %)	7548 bp (5.80 %)	595 bp (1.29 %)
Retroelements	7 (6,78 %)	7 (7,52 %)	9 (4,65 %)	0
LINEs:	0	1 (0,70 %)	2 (0,16 %)	0
L1/CIN4	0	1 (0,70 %)	2 (0,16 %)	0
LTR elements:	7 (6,78 %)	6 (6,82 %)	7 (4,49 %)	0
Ty1/Copia	1 (1,09 %)	1 (0,24 %)	1 (0,55 %)	0
Gypsy/DIRS1	6 (5,60 %)	5 (6,58 %)	6 (3,94 %)	0
DNA transposons	0	0	2 (0,10 %)	0
Simple repeats:	18 (0,67 %)	17 (0,94 %)	18 (0,69 %)	11 (0,98 %)
Low complexity:	1 (0,03 %)	3 (0,25 %)	7 (0,36 %)	2 (0,31 %)

### Genomic DNA capture and gene model generation

At the same time as the BAC library screening, we conducted a gene capture procedure using maritime pine haploid DNA [25]. Genomic DNA captured obtained using the SureSelect kit was sequenced in 454/Roche, and a total of 2,036,142 captured raw reads with an average size of 769 nt were cleaned with SeqTrimNext, producing 1,942,057 useful reads. These reads were assembled with MIRA3, yielding a total of 144,707 contigs and 305,396 “debris” reads. The contigs served to reduce the sequence space and to provide longer consensus sequences to facilitate the building of the gene model.

**Fig. 2:**
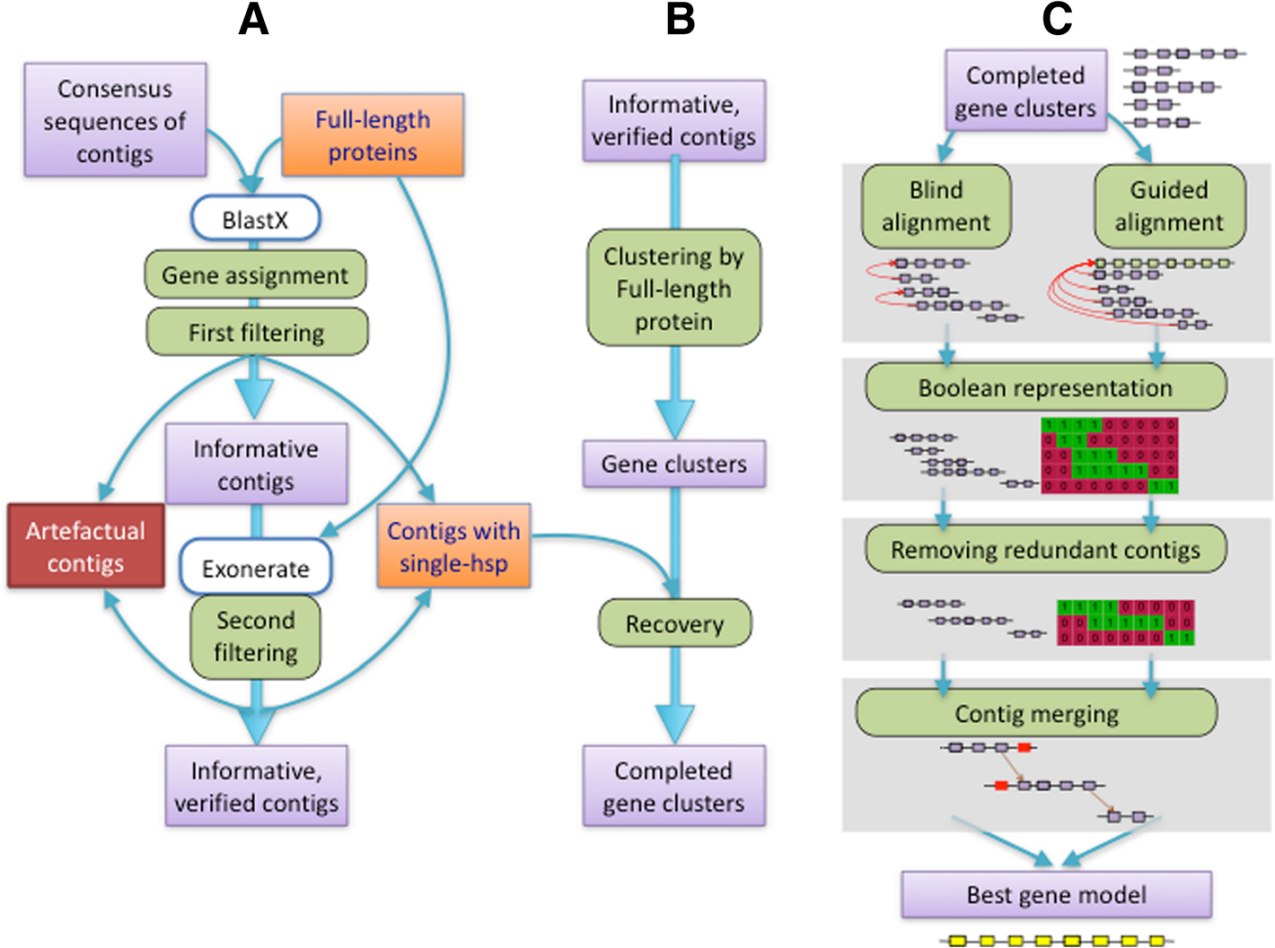
Flow chart for the Gene Assembler pipeline. **a** Gene assignment and contig filtering. **b** Contig clustering gene. **c** Gene model building. See text for details.

The GeneAssembler pipeline was developed to build the most complex gene model that could code for each of the 866 selected full-length cDNAs from the pine transcriptome described in [20]. Starting with 144,707 contigs, the following steps were included: (i) gene assignment and contig filtering (Fig. 2a), (ii) contig clustering by gene (Fig. 2b) and (iii) gene model building to produce a gene model for the selected full-length proteins (Fig. 2c).

The best gene model is as follows: (i) the one that recovers more full-length seeding sequences; (ii) the one with the lowest overlap percentage of contigs at the exon level; and (iii) the one with the lowest fragmentation index, that is, the one obtained from the fewest number of contigs. The GeneAssembler gene model for each full-length protein was saved in a gff3 file, which included the contigs. Additionally, a FASTA file was generated with the gene model sequences and an index relating each contig with its full-length protein.

Genomic sequences were recovered for the 866 maritime pine genes selected to design the probes, and this recovery was independent of the use of a reference genome for building the gene model. Accordingly, the reconstruction that was done without a reference genome was selected as it represents the highest mean recovery of each protein, approximately 82 %, and the lowest sequence redundancy. Most of the gene models with a high recovery percentage had between one to five exons, and particularly for those with one and two exons, there were many gene models with a recovery rate above 50 %. In addition, there were 20 gene models with a recovery rate over 100 %, but these were considered to be incorrect due to the presence of sequence repeats, which the algorithm was unable to handle. All of the generated contigs and models are available in the Pine Gene Capture database (PGC, http://www.scbi.uma.es/pgc/).

**Fig. 3:**
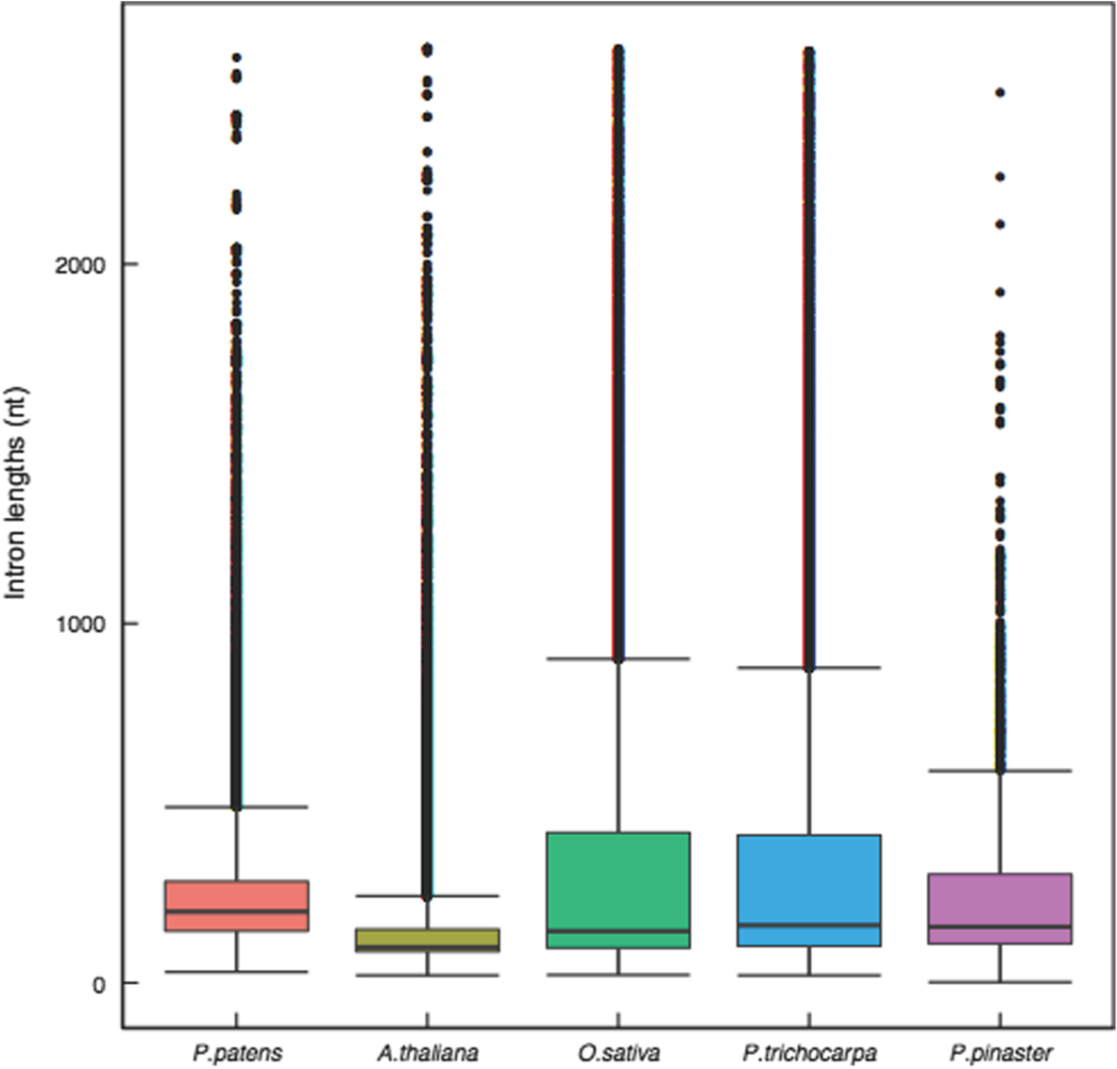
Distribution of individual lengths of introns smaller than 2600 nt using the 866 reconstructed gene models in *P. pinaster* and their orthologs from P*. patens, A. thaliana, O. sativa* and *P. trichocarpa.* The box plot includes the median values as well as outlier lengths.

Because there is increasingly more information available about plant genomes, it is possible to make broad comparisons among them. Although it has been suggested that organisms with small genomes have smaller introns, studies of angiosperms indicate that this is not necessarily true in plants [26, 27]. We performed an analysis of the size of the introns for our 866 reconstructed maritime pine models and compared the results to homologs in the moss *Physcomitrella patens* and three well-characterized angiosperm genomes*: Arabidopsis thaliana*, *Oryza sativa* and *Populus trichocarpa* (Fig. 3). The average length of the individual introns (in bp) was 197, 98, 143, 159 and 155 for *P. patens, A. thaliana*, *O. sativa, P. trichocarpa* and *P. pinaster,* respectively. The average intron length did not vary significantly between *P. pinaster* and the other four models chosen for this study (Fig. 3). However, the intron lengths were more heterogeneous in *O. sativa* and *P. trichocarpa* than in the other three species considered, at least for the 866 genes included in this study. Maritime pine did not appear to have a significantly larger intron.

### Comparison of BAC and gene capture approaches to defining maritime pine gene models

To further explore the quality of the genomic sequences derived from the gene capture approach, the structures of the *AS*, *SuSy* and *XET* genes were compared to those derived by BAC clone sequencing.

**Fig. 4:**
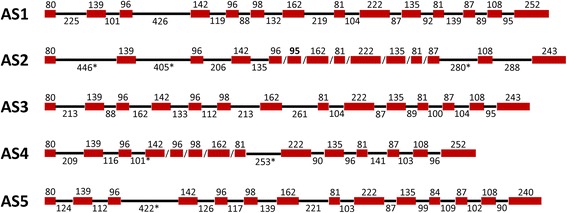
Exon/intron gene model for the *AS* family in maritime pine. The *red boxes* are exons and the black lines introns. The corresponding size in nucleotides is indicated. The *asterisks* indicate introns with incomplete sequences.

Tables 1 and 2 show the distribution of exons and introns, respectively, for the *AS* gene obtained by BAC sequencing and that obtained by gene capture. After the analysis of the gene capture contigs, four different *AS* gene models were found to be similar to *AS1*, including itself; the new sequences were named *AS3*, *AS4* and *AS5*. While cDNA contigs for the *AS3* and *AS4* genes were found in SustainPineDB (sp_v3.0_unigene3120 and sp_v3.0_unigene97582 respectively) [20], no contigs for *AS5* were found in SustainPineDB or Genbank. However, the genomic sequences for the *AS5* ortholog are present in the *Pinus taeda* genome [*PtAS5*, Congenie:lcl|scaffold870050.1]. Figure 4 presents the structures of the five *AS* genes of *P. pinaster*, with their relative exon/intron sizes*.* The *AS1* and *AS3* gene structures were completed, and the *AS5* gene sequence was almost completed except for a part of the third intron. However, the *AS4* structure was not completed due to a lack of information for the 3^rd^ to the 8^th^ introns. The gene capture assay also included probes for *AS2,* and the sequence was independently analyzed with respect to *AS1*. No crossovers were observed between *AS1* and *AS2* in the gene model building process, but the gene model for *AS2* was incomplete as it lacked information for the 5^th^ to 11^th^ introns. The gene capture model that was generated fits to the *AS3* gene as well as to *AS1* and *AS5*. Although the comparison of the gene capture and BAC data indicates that the exon lengths and distributions from both methods agree well, the structure of the *AS* gene in the BAC clone was identical to *AS1* gene model based on the gene capture. This provides additional confirmation that the isolated BAC contained a genomic fragment encoding the maritime pine AS1 protein [28].

The gene capture model for the *SuSy* gene agreed well with the structure of the BAC clone. The maritime pine model is closer to the *SuSy2* gene from *Arabidopsis* and *Populus* with very well conserved number, length and exon position (Additional file 2: Table S1). Only exons 7 and 15 displayed small differences of 6 and 27 nt, respectively. The combination of BAC sequencing and gene capture allowed us to complete the first exon sequence, which can be considered an additional advantage of combining the two strategies.

The BAC clone and the gene capture model displayed very similar intron sizes; even intron 8, which contained a gap in the BAC assembly, was completed using the gene capture data (Additional file 3: Table S2). Although the distribution and length of exons was well conserved, an increase in the variability of the intron size was observed, which was similar to what was found for the *AS* gene family. In this case, the average size of the introns of the maritime pine gene was higher than for those genes of the other two species included in the comparison.

In the third part of this study, building the *XET* models, we did not include poplar genes, as we did for the *AS1* and *SuSy* genes. The *XET*-related gene family is very large in plants, and as the poplar genome recently underwent a whole genome-wide duplication [29], the number of *XET* genes to compare to find the closest model to the pine gene would be very large. Therefore, considering the larger size of the gene family, we could not obtain a single model through gene capture. Instead, the two potential gene models that were generated are listed in Additional file 4: Table S3.

### Gene capture of regulatory regions

An additional aim of this study was to test if the gene capture methodology could be extended to recover unknown sequences of the 5´end of the genes so that these sequences could be used: i) for a genome walking approach to obtain the promoter sequence or ii) directly for functional studies using the proximal promoter region of the genes.

**Fig. 5:**
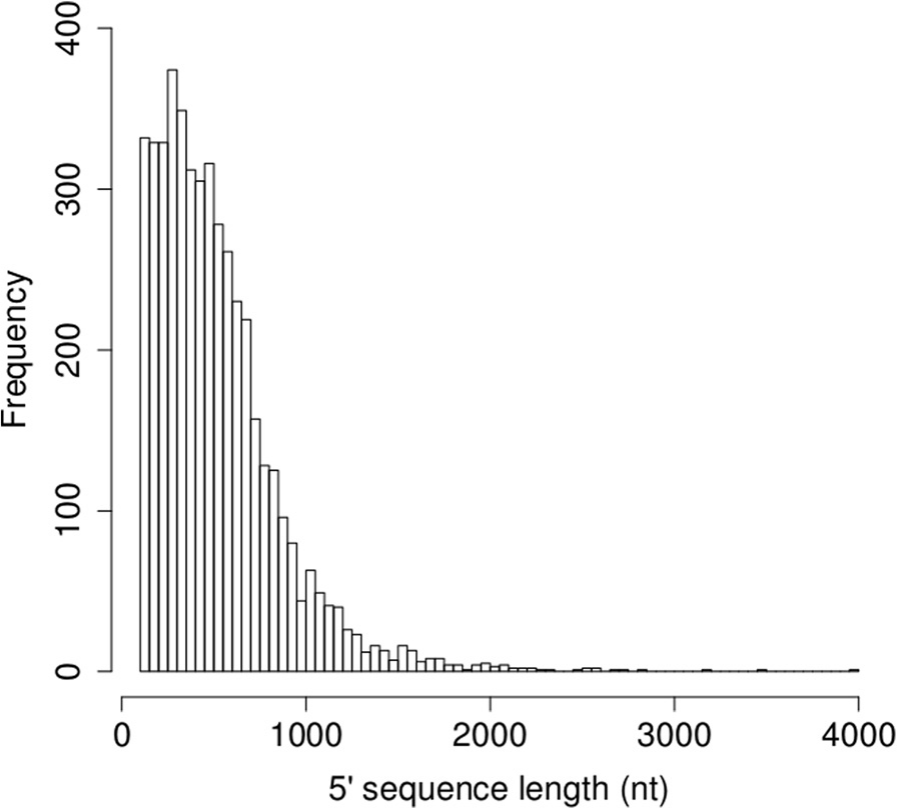
Frequency distribution of contigs containing the 5´upstream region of the gene models generated. The sequence length recovered is expressed in nucleotides. The distribution was built using the contigs containing the first exon where the amino acid 10 or previous is present and where from the beginning of the first exon have at least 100 nt upstream.

To obtain the 5´end of the captured genes, contigs containing the first exon and having at least 100 nt upstream of the first exon were selected. Based on these standards, we obtained the frequency distribution of the contigs containing a noticeable upstream region of the genes (Fig. 5). The estimation from 866 gene models used in the gene capture approach was that 737 gene models contained a noteworthy 5´region sequence. Of those, 480 had a 5´region ranging 500 to 1000 nucleotides.

To validate these results, the 5´upstream sequences of three genes included in our gene capture study were compared to the corresponding gene promoter sequences that had been previously cloned and functionally characterized [30, 31]. For the glutamine synthetase (*GS1a)* gene, 518 nucleotides of the sp_v1.1_unigene26377 gene capture model overlapped with the GenBank promoter sequence [GenBank:AJ225121] (Additional file 6: Figure S2); 907 nucleotides for the phenylalanine ammonia lyase (*PAL)* gene promoter [GenBank:HE866754] overlapped with the sp_v1.1_unigene15094 model (Additional file 7: Figure S3); and 630 nucleotides for the prephenate aminotransferase (*PAT)* gene promoter [GenBank:HE866755] matched the unigene all_rep_c3941_PAT gene capture model (Additional file 8: Figure S4).

## Discussion

In this study, we present a strategy to generate maritime pine gene models, confirmed by the structure of available BAC clones, which demonstrates that gene capture is a powerful technology for establishing gene structures in species without reference genomes, such as the maritime pine.

### Maritime pine gene structure

Generally, it is well accepted that a positive relationship exits between genome size and intron lengths in eukaryotes. In conifers, which have large genomes ranging from 18 to 35 Gbp, longer introns can contribute to large genome sizes [12]. In this study, we performed a deep comparison of exon/intron distribution for three pine genes against the *Arabidopsis* and poplar sequences from the databases.

**Fig. 6:**
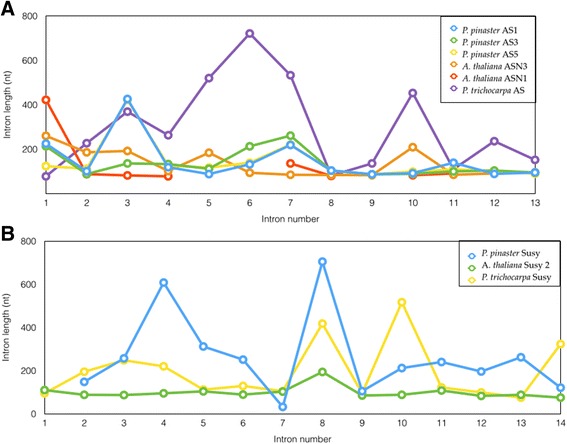
Representation of gene intron size: (**a**) *AS* genes *in P. pinaster, A. thaliana* and *P. trichocarpa*; (**b**) *SuSy* gene from *P. pinaster*, *A. thaliana* and *P. trichocarpa*. On the X axis is showed the position of the introns and on the Y axis is showed the length of introns in nucleotides.

The intron lengths for the *AS1* gene (Table 2) are similar to those described for *ASN1* and *ASN3* in *Arabidopsis,* with the exception of introns 3 and 9, but none were greater than 426 bp, the length of intron 3 in the *AS1* gene. In fact, when the *AS1* intron sizes were compared to the poplar gene models, the medium intron size was larger in poplar than in *Arabidopsis* or pine (Fig. 6a). When intron size is compared in the structure of the *SuSy* gene, the distribution is also well conserved among the pine BAC, *Arabidopsis SuSy2* and poplar POPTRDRAFT_830445 genes. However, intron length is most variable among the three species, with longer introns for the maritime pine *SuSy* gene (Fig. 6b). For the *XET* gene, no significant differences were found in the size, distribution, number or length of the intron when compared to the *Arabidopsis* gene models. Moreover, a broad comparison of the intron sizes for the 866 gene models generated in this study (Fig. 3) showed that the medium intron size is in the range of that observed for other angiosperms models [18] (for more details, see gene models in the Pine Genome Capture Database PGCD: http://www.scbi.uma.es/pgc/).

The second feature contributing to the large conifer genomes is retrotransposon expansion, which contrasts with what has been described for large angiosperm genomes, where gene duplications and, polyploidization, as well as retrotransposons expansion, are the most common features contributing to genome size [32, 33]. Thus, retrotransposon expansion can be of primary importance in explaining genome size in *Pinus* species [34]. We analyzed the presence of retroelements in the BAC clones included in this study by comparing two available BACs containing the *AS1* and *SuSy* genes in *P. glauca*.

In terms of number/percentage, the LTR and Gypsy retrotransposons are most abundant in the *SuSy* and *AS1* BACs from *P. glauca* and *P. pinaster* (Table 3). These findings suggest that retrotransposon expansion is a reasonable hypothesis to explain the large genome size in *P. pinaster*, as has been proposed for other conifers [34, 35]. However, much more data on the retrotransposon families of maritime pine are needed to confirm this hypothesis.

### Gene capture results are consistent with BAC clone structures; this outcome can be extended to other frames

The algorithm developed for building gene models in this work has been used with plants as target organisms, but it is not restricted to them, its use with gene capture data derived from other organisms is possible.

A total of 866 gene models were obtained with our in-house bioinformatics pipeline (GeneAssembler), with a median recovery percentage of 82 % from the original full-length sequence. However, there were several constraints, such as complex gene families, the presence of pseudogenes and the lack of a reference genome, that limited the final results. The algorithm designed in this study can address these problems, correctly resolving the gene models. It is also necessary to note is that the guided recovery using genes from model organisms, such as *A. thaliana*, *P. trichocarpa*, *O. sativa* or *P. patens,* could be useful when the coding sequence is incomplete. However, if enough transcript sequence information is available, the model can be built without any reference, which can be justified by taking into account that the contigs used to build the gene model come from the pool of single-hsp contigs. This pool has no defined exon-intron contig structure, and the contigs can be either exon fragments or putative pseudogenes.

The pipeline was also able to provide sequences for the 5´upstream region of most genes (85 %), and furthermore, 55 % of the gene models have at least one contig containing 500 to 1000 bp of the regulatory region of the gene. These findings indicate that the pipeline can be tuned to find a substantial portion of the gene promoter regions. These results validate the use of gene capture as a technology to provide information about the promoter region of genes in species where reference genomes are not available. This genomic information can be used *in silico* studies or to obtain the promoter region for functional studies of a significant number of maritime pine genes.

### Exploring the organization of maritime pine gene families

**Fig. 7:**
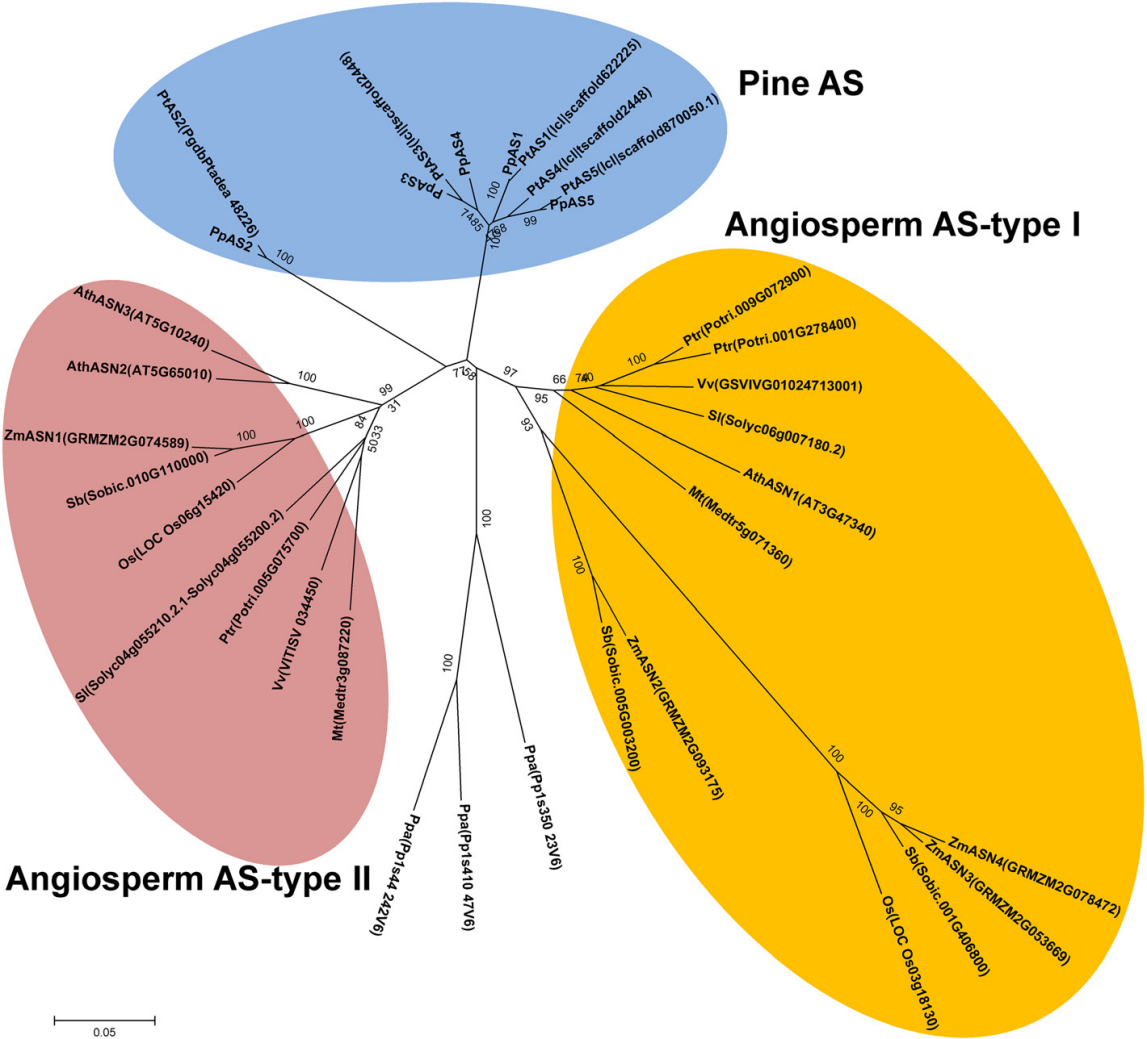
Phylogenetic tree of the deduced protein sequences of plant genes encoding asparagine synthetase (AS). The optimal tree with the sum of branch length = 2.28234152 is shown. The tree is drawn to scale, with branch lengths in the same units as those of the evolutionary distances used to infer the phylogenetic tree. The following representative members of the asparagine synthetase (*AS*) are included in the tree: *Arabidopsis thaliana* [*AthASN1*, Phytozome:AT3G47340; *AthASN2*, Phytozome:AT5G65010; *AthASN3*, Phytozome:AT5G10240), *Medicago truncatula* [Phytozome:Medtr5g071360; Phytozome:Medtr3g087220], *Oryza sativa* [Phytozome:LOC_Os06g15420; Phytozome:LOC_Os03g18130], *Physcomitrella patens* [Phytozome:Pp1s410_47V6; Phytozome:Pp1s350_23V6; Phytozome:Pp1s44_242V6], *Pinus pinaster* [*AS1*, GenBank:ADU02856; *AS2*, Genbank:ADK13052; *AS3*, PGC:geneCapture_all_rep_c7631; *AS4*, SPDB:sp_v3.0_unigene97582/sp_v3.0_unigene8248; *AS5*, PGC:geneCapture_all_rep_c8956/geneCapture_all_rep_c1052], *Pinus taeda* [*PtAS1*, Congenie:lcl|scaffold622225; *PtAS2*, Congenie:PgdbPtadea_48226; *PtAS3*, Congenie:lcl|tscaffold2448; *PtAS4*, Congenie:lcl|tscaffold2448; *PtAS5*, Congenie:lcl|scaffold870050.1], *Populus trichocarpa* [Phytozome:Potri.005G075700; Phytozome:Potri.009G072900; Phytozome:Potri.001G278400], *Sorghum bicolor* [Phytozome:Sobic.005G003200; Phytozome:Sobic.001G406800; Phytozome:Sobic.010G110000], *Solanum lycopersicum* [Phytozome:Solyc06g007180.2; Phytozome:Solyc04g055210.2.1- Phytozome:Solyc04g055200.2], *Vitis vinifera* [Phytozome:GSVIVG01024713001; Phytozome:VITISV_034450], *Zea mays* [*ZmASN1*, Phytozome:GRMZM2G074589; *ZmASN2*, Phytozome:GRMZM2G093175; *ZmASN3*, Phytozome:GRMZM2G053669; *ZmASN4*, Phytozome:GRMZM2G078472]

Asparagine synthetase plays a specific role in nitrogen assimilation and storage in pine trees [28, 36]. The data derived from gene capture described in this manuscript revealed that this enzyme is encoded by a small gene family in maritime pine with five members: *AS1, AS2, AS3, AS4* and *AS5*. We also found their counterparts in the *P. taeda* genome (Fig. 7). It is remarkable that this approach allowed us to obtain the sequence of a new gene, *AS5*, which had not been previously identified by transcriptomic analyses. The nucleotide sequences of the five genes (Additional file 9: Figure S5) and the deduced AS polypeptides (Additional file 10: Figure S6) are well conserved, with the highest variability located at the 3´end or the carboxy- terminus of the protein. The *AS1, AS3, AS4* and *AS5* genes/proteins are more closely related to each other than to *AS2*.

Phylogenetically, the angiosperm *AS* genes can be grouped into two classes: *ASI* and *ASII* [37, 38]. The *AS* genes belonging to *ASII* class generally have 13 introns while the *ASI* class lacks intron five [38]. A phylogenetic tree was generated using the deduced amino acid sequences of the five *AS* pine genes from *P. pinaster* and *P. taeda* in addition to the *AS* sequences from *Physcomitrella patens* and different angiosperms used in previous *AS* phylogenetic analysis [38] (Fig. 7). Until now, the gymnosperm *AS* genes were considered to belong to the *AS* class II group [39]. But it can be concluded from our phylogenetic analysis that the conifer AS proteins do not belong to either *AS* class I or class II, which had been previously described for angiosperms. As shown in Fig. 7, the pine AS proteins group together separately from either of the ASI or ASII angiosperm classes. The models that are phylogenetically closer to angiosperms are the class II *ASN3* gene from *Arabidopsis* and the class II Potri.005G075700 gene from poplar, which were included in our comparative study of exon/intron *AS* gene organization. It has previously been suggested that the angiosperm *AS* class II genes are closer to the ancestral *AS* gene [38], and our results support this hypothesis because all conifer *AS* genes conserve the 13 introns in their structure. Moreover, the conifer *AS* genes are closer to the bryophyte *P. patens* at sequence level.

The *AS* BAC clone sequenced in this work contains the genomic sequence of the *AS1* gene, but due to the close similarity among the *AS1, AS3* and *AS5* pine genes (Tables 1 and 2, Fig. 4, Additional file 9: Figure S5 and Additional file 10: Figure S6), the model we obtained using the designed algorithm was predominantly *AS3.* A thorough examination of the contigs recovered by gene capture is included in Additional file 11: Table S6, and it illustrates that numerous contigs could be included in the *AS3* instead of the *AS1* model in this case. There were mainly truncated sequences, or intron-lacking sequences, that can be considered to be pseudogenes of *AS3*, and this indicates that the presence of numerous pseudogenes derived from a single gene can bias the results of this type of gene capture experiment. In the early stages of our study, we included this gene model because we thought it would be a good example of a pine gene belonging to a family with relatively few members to the *XET* gene and its family composition. In addition, the *AS* gene has a relatively large number of exons compared to the structure of the *XET* gene, which has fewer exons that can be used to detect differences in sequence recovery. However, we have found that this additional information related to the numerous pseudogenes in the *AS* family highlights the complexity of the maritime pine genome.

## Conclusions

The comparison of the gene models produced by both approaches revealed that (i) processing the gene capture data with GeneAssembler was highly successful with 82 % of the gene models recovered even when a reference genome was not available. (ii) Successful and useful gene models can be obtained using probes designed from cDNAs; inference from of a single gene model would be limited in complex gene families with many members for which supplementary information would be required. (iii) Gene capture can serve to fill gaps in the gene structure established by BAC sequencing, as is the case with the *SuSy* gene model included in this study. (iv) Studying the gene models of different members belonging to the same family (e.g., *AS*) creates new possibilities for facilitating intra- and inter- comparative studies aimed at understanding function in the light of evolution.

## Methods

### PCR-based screening of a pooled BAC library

A 0.8x coverage pooled BAC library was previously prepared [22] as 83 glycerol stocks of *E. coli* pools, and each pool contained approximately 4000 distinct clones with an average size of 107 kbp. DNA of BAC pool was prepared by a modified alkaline lysis method using a previously described protocol [40].

For the primary PCR screening, specific genomic sequences of asparagine synthetase (*AS*), sucrose synthase (*SuSy*) genes or xyloglucan endotransglycosylase (*XET*) were amplified using primers designed against the available cDNA sequence (Additional file 12: Table S5). The PCR profile used for screening the pool library was as follows: 20 s denaturing at 94 °C, 45 s annealing at 60 °C and 45 s elongation at 72 °C. This temperature profile was repeated for 35 cycles, and putative positive PCR product pools were on sequenced a CEQ 8000 automated capillary sequencer (Beckman Coulter, Barcelona Spain). After this, the original cell pools were titrated to determine the appropriate dilution to obtain ~7000 colonies per Qtrays Genetix plate (24.2 × 24.2 cm), and the different clones were then individualized in 36 × 384-well plates using a QPIX2 ® (Genetix).

For the secondary screening, high-density filters were prepared on 22.2 × 22.2-cm nylon membranes using a 96-pin gridding head on the Genetix QPIX2 ® robot. Each BAC filter was gridded to 6 rows × 4 columns in a single spot and distributed in six different fields. Membranes were incubated overnight at 37 °C with the colony side facing up on LB/chloramphenicol (12.5 μg/ml) plates, and processing and hybridization of the filters was done as recommended in the QPix manual. [^32^P]-labeled specific genomic sequences were used as probes. The hybridized membranes were exposed to a phosphorimaging screen (Fuji Imaging plate BAS-MS 2040) for 24 h at room temperature and scanned using an FLA-7000 Imaging System (Fujifilm). Using a grid map, the pattern of hybridization allowed us to identify the 384-well plate(s) and the plate address(es) of the positive clone(s).

For the subsequent steps, the cells of the positive clones were recovered and plated on LB agar with 12.5 μg/ml chloramphenicol, 90 μg/ml IPTG and 90 μg/ml X-GAL. After a second round of PCR and sequencing, the presence of a specific genomic sequence was confirmed. The detailed workflow protocol is available in Additional file 1: Figure S1.

### BAC DNA Sequencing library preparation and 454 parallel sequencing

The BAC insert size was estimated by pulse field gel electrophoresis (FIGE, Bio-rad Lab. Inc.). The 454 sequencing library was prepared with 6 μg of the BAC plasmid DNA using shotgun and standard long, paired-end sequencing kits, according to the specifications of Roche, the manufacturer. Sequencing libraries were quantified with 2100 BioAnalyzer (Agilent), processed by emulsion PCR and sequenced on a 454/Roche GS FLX as described in the manuals (Roche Diagnostics). The 24 L12 (*AS*), 25 M3 (*XET*) BACs libraries, were respectively sequenced in paired-end and shotgun pools using MIDs loaded in a 1/2 region, and BAC 26C15 library (*SuSy*) was sequenced in paired-end pools in a 1/4 region of 70 × 75 Picotiterplate (PTP). The pair-end BAC reads, which had been preprocessed by SeqTrimNext, were assembled using Newbler version 2.3 with the default parameters.

### Capture array gene selection

The 91,086 full-length unigenes predicted from the *P. pinaster* transcriptome assembly from SustainPineDB [20] were used to select the genes for the DNA capture array. The full-length unigenes were filtered based on their GO terms [41], and the KEGG pathways [42] annotations mainly focused in metabolism. Because some unigenes contained annotations from several functions, this filter resulted in 1462 unigenes. Additionally, to reduce redundancy, the unigenes sharing the same ortholog from UniProt [43] were filtered to get only one gene per ortholog, reducing the selected number of unique unigenes to 1026. BLAST [44] best hits were used to assign the orthologous genes.

After the unigene selection was done, a better assembly of *P. pinaster* transcriptome was performed and published [20], so we searched for the genes from the 1026 unigenes in the SustainPine assembly version 1.1 (http://www.scbi.uma.es/sustainpinedb/) using BLAST. Finally, 866 unigene sequences were selected to be included in the DNA capture array. As described previously, we added 3 genomic sequences from cloned BACs as control sequences, the asparagine synthetase [BAC at GenBank: KP172187] from 5’ UTR to the stop codon, a clone containing the sequence of a gene encoding a sucrose synthase [BAC at GenBank: KP172194] and a clone containing the xyloglucan endotransglycosylase [BAC at GenBank: KP172185]. The distribution of the unigenes used for the capture array in functional categories is shown in Additional file 13: Figure S7.

### DNA capture array design

A Ruby custom script was used to design the probes for an Agilent SureSelect DNA Capture Array, and it was optimized to fit the 866 genes selected (see *Capture array gene selection*). In total, 56,667 probes of 120 nt in length were designed, and the probe density was increased in the first third of the transcript sequences (including the 5’UTR). In this way, all of the genes were represented in the array with 6x coverage in the first third of the gene sequence (using a tilling distance of 19 nt) and 4x coverage in the other two thirds (using a tilling distance of 29 nt). On average, 65 overlapping probes represented each gene, and they were uploaded to the Agilent eArray website (https://earray.chem.agilent.com/earray/) for the production of the array.

### Haploid genome DNA preparation

Maritime pine cones were collected from Oria 6, a genotype of *P. pinaster* Aiton from the natural population Sierra de Oria (Almería, Spain), selected based on its response to extreme drought conditions. Cones were surface sterilized with 96 % ethanol for 20 min and air-dried in a laminar flow cabinet before seed isolation. Haploid megagametophytes were isolated from sterilized seeds for tissue culture establishment [25]. For sequence capture, the *P. pinaster* A5 callus haploid line derived from the megagametophyte tissue was used. The DNA was extracted from the calli using a DNeasy Plant Mini Kit (Qiagen) and it was quantified with a Quant-it PicoGreen dsDNA Assay Kit (Invitrogen). The quality was assessed with an Agilent 2100 Bioanalyzer.

### Targeted capture and 454 parallel sequencing

Haploid genomic DNA from *P. pinaster* was captured using the Agilent SureSelect Target Enrichment System following the manufacturer´s protocols with minor modifications. Two micrograms of this DNA were fragmented to 1.5 kb in size and purified by gel extraction using a MinElute Gel extraction kit (Qiagen), and the quality of fragmentation and purification was assessed with an Agilent 2100 Bioanalyzer. Fragment ends were repaired, and RL adaptors (Roche) were ligated to the fragments, and the resulting adapter-ligated sample was purified using Agencourt AMPure XP beads (Beckmann Coulter). The DNA library was amplified by PCR and captured by hybridization at 65 ^o^C for 24 h with the biotinylated RNA library “bait” (Agilent). Bound genomic DNA was purified with streptavidin-coated magnetic Dynabeads (Invitrogen) and re-amplified. Stratagene Herculase II enzyme (Agilent) was used for both PCR reactions, and the resulting captured library was purified using Agencourt AMPure XP beads (Beckmann Coulter) and was assessed with the Agilent 2100 Bioanalyzer. Finally the captured library was sequenced on Roche GS-FLX+ using a two-region gasket according to the manufacturer´s protocols.

### Roche 454 data processing

The reads were preprocessed using the SeqTrimNext pipeline (http://www.scbi.uma.es/seqtrimnext) [45], which is available from the Plataforma Andaluza de Bioinformatica (University of Malaga, Spain). Low quality sequences, linkers, adaptors, vector fragments, organelle DNA, and contaminated sequences were removed, and the longest informative part of the read was retained, discarding sequences below 40 bp.

### Data assembly and gene recovery criteria

Useful reads were assembled by the MIRA assembler, version 3 [46], to obtain the contigs for building the gene models. Full-length transcripts and the protein sequences were predicted using Full-Lengther Next [47] based on SustainPine 1.1 database [20] information.

The captured fragments were identified, and the gene models were generated using our own bioinformatic pipeline (GeneAssembler), which can be downloaded and installed on any Unix/Linux-based computer as a Ruby gem: https://rubygems.org/gems/gene_assembler (for a detailed description of the GeneAssembler pipeline see Additional file 14).

The gene sequences from *Arabidopsis thaliana*, *Populus trichocarpa*, *Oryza sativa* and *Physcomitrella patens* were downloaded from Phytozome 9.1 [48] to improve gene recovery, and an ortholog search was performed to enhance the gene model building strategy. A Blast X (by default parameters) [49] with the full-length proteins was performed against each set of genes. All gene matches were considered to be putative orthologs, which means that for each match, the exon-intron coordinate was retrieved.

### Sequence alignment and phylogenetic analysis

The sequences used for alignments and phylogenetic trees were obtained in Phytozome database (http://phytozome.jgi.doe.gov) except for *P. taeda* that were obtained from Congenie database (http://congenie.org/). *P. pinaster* AS1 [GenBank:ADU02856]; and AS2 [GenBank:ADK13052] protein sequences were obtained from GenBank at the NCBI. For *P. pinaster* AS3 [PGC:geneCapture_all_rep_c7631] and AS5 [PGC:geneCapture_all_rep_c8956/geneCapture_all_rep_c10521 we used sequences obtained in the course of this work and deposited in the Pine Gene Capture database (PGC, http://www.scbi.uma.es/pgc/). Finally for *P. pinaster* AS4 [SPDB: sp_v3.0_unigene97582/sp_v3.0_unigene8248] we used the sequence obtained from our transcriptomic database SustainPineDB [20] (http://www.scbi.uma.es/sustainpinedb/).

The CLUSTALW program was used for sequence alignments [50]. The phylogenetic tree was constructed with full-length AS amino acid sequences using the neighbor-joining method [51] with 1000 bootstrap replications. The evolutionary distances were computed using the JTT matrix-based method [52] and are in the units of the number of amino acid substitutions per site. The rate variation among sites was modeled with a gamma distribution (shape parameter = 1). All ambiguous positions were removed for each sequence pair. The positions not presented in all the sequences were eliminated. Finally there were a total of 509 positions in the final dataset. All of these analyses were conducted in MEGA6 [53].

### Availability of supporting data

The bioinformatic pipeline (GeneAssembler) used for generating gene models, can be downloaded at: https://rubygems.org/gems/gene_assembler. All of the generated contigs and models are available in the Pine Gene Capture database (PGC, http://www.scbi.uma.es/pgc/). The phylogenetic data can be found at http://purl.org/phylo/treebase/phylows/study/TB2:S18787. Other supporting data of this article are included as additional files

## Additional files

Additional file 1: Figure S1. Workflow overview followed to isolate and sequence maritime pine BAC clones. (A) Primary PCR screening of pools of the BAC library using specific oligonucleotides against *XET* cDNA. (B) Plating of cells of the positive pools on 24.2 X 24.2 cm plates. (C) The single clones of the pool were individualized in 36 X 384 plates using a QPix and grown orderly gridded in high-density filters. (D) The replica filters containing the ordered single clones of each pool were hybridized with cDNA ^32^P-labeled specific probes and exposed to a phosphorimaging screen. (E) Secondary PCR screening was performed on single clones to isolate positives. (F) BAC DNA isolation of positives to prepare a 454 library either for shotgun or paired-end sequencing. (TIFF 6431 kb) Additional file 2: Table S1. Exon length comparison between *SuSy* BAC clone from *P. pinaster* and *SuSy* from two angiosperm plants. The first exon is lacking in the BAC clone. The gene capture model is also included (DOCX 19 kb) Additional file 3: Table S2. Intron length comparisons between *Susy* BAC clone from *P. pinaster* and *Susy* from two angiosperm plants. The intron I8 in the BAC clone contains a gap. The gene capture model is also included (DOCX 20 kb) Additional file 4: Table S3. Exon length comparison between the *XET* BAC clone from *P. pinaster* and two *XET* genes from *Arabidopsis thaliana*. The two gene capture models closest to the BAC clone are also included. (DOCX 18 kb) Additional file 5: Table S4. Intron length comparison between the *XET* BAC clone from *P. pinaster* and two *XET* genes from *Arabidopsis thaliana*. The two gene capture models closest to the BAC clone are also included. (DOCX 17 kb) Additional file 6: Figure S2. Alignment of the *GS1a* gene promoter [GenBank:AJ225121], to the gene capture *GS1a* gene 5´upstream region (TIF 245 kb) Additional file 7: Figure S3. Alignment of the *PAL* gene promoter [GenBank:HE866754], to the gene capture *PAL* gene 5´upstream region (TIF 389 kb) Additional file 8: Figure S4. Alignment of the *PAT* gene promoter [GenBank:HE866755], to the gene capture *PAT* gene 5´upstream region. (TIF 332 kb) Additional file 9: Figure S5. Nucleotide alignment of maritime pine *AS* cDNAs. (TIF 533 kb) Additional file 10: Figure S6. Amino acid alignment of maritime pine AS protein (TIF 460 kb) Additional file 11: Table S6. Summary of *AS* contigs obtained in the gene capture and used to build the *AS* model. (XLS 138 kb) Additional file 12: Table S5. Primers used in PCR for validation of positive BAC clones in the BAC library screening. (DOCX 39 kb) Additional file 13: Figure S7. Functional categories by GO terms for the full-length unigenes used in the capture approach (TIF 269 kb) Additional file 14: Detailed description of the GeneAssembler pipeline (DOCX 124 kb)
