# Supplementary figures and images for: Establishing gene models from the Pinus pinaster genome using gene capture and BAC sequencing
Source: BMC Genomics. 2016 Feb 27;17:148. doi: 10.1186/s12864-016-2490-z (PMC4769843; doi:10.1186/s12864-016-2490-z)

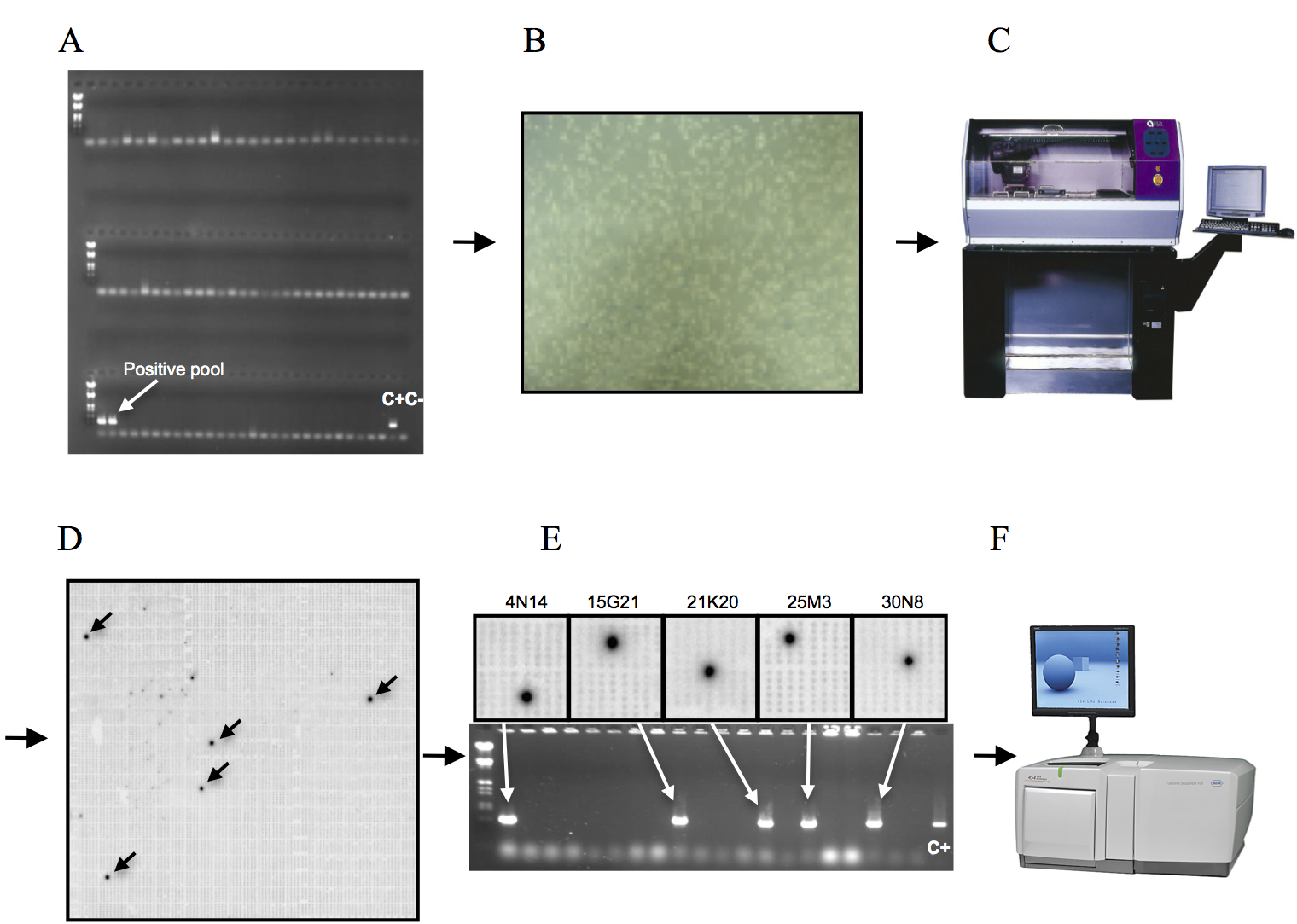

Supplement: Additional file 1: Figure S1. — Workflow overview followed to isolate and sequence maritime pine BAC clones. (A) Primary PCR screening of pools of the BAC library using specific oligonucleotides against XET cDNA. (B) Plating of cells of the positive pools on 24.2 X 24.2 cm plates. (C) The single clones of the pool were individualized in 36 X 384 plates using a QPix and grown orderly gridded in high-density filters. (D) The replica filters containing the ordered single clones of each pool were hybridized with cDNA 32P-labeled specific probes and exposed to a phosphorimaging screen. (E) Secondary PCR screening was performed on single clones to isolate positives. (F) BAC DNA isolation of positives to prepare a 454 library either for shotgun or paired-end sequencing. (TIFF 6431 kb) [file 12864_2016_2490_MOESM1_ESM.tiff]

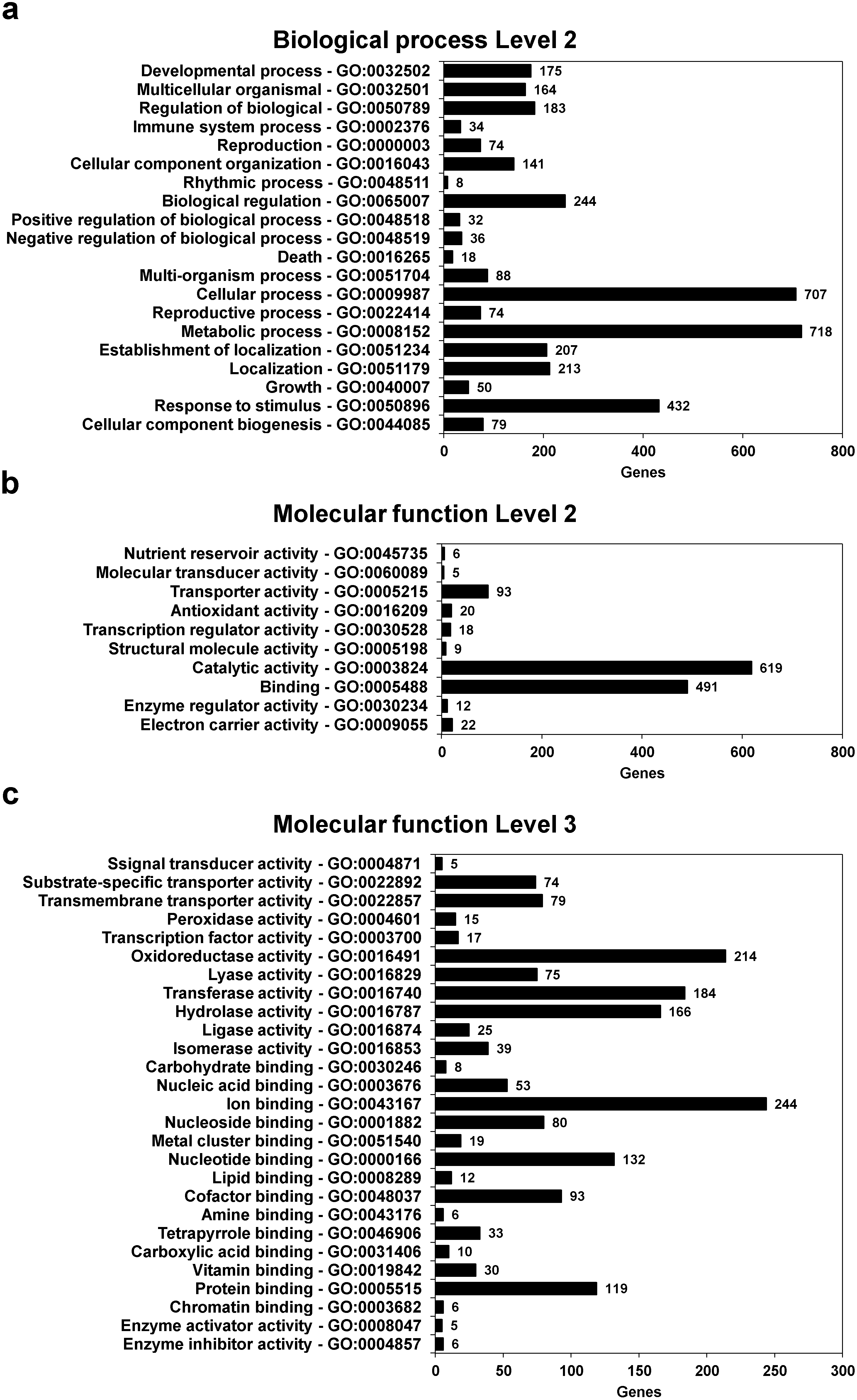

Supplement: Additional file 13: Figure S7. — Functional categories by GO terms for the full-length unigenes used in the capture approach (TIF 269 kb) [file 12864_2016_2490_MOESM13_ESM.tif]
