# Supplementary material for: Establishing gene models from the Pinus pinaster genome using gene capture and BAC sequencing
Source: BMC Genomics. 2016 Feb 27;17:148. doi: 10.1186/s12864-016-2490-z (PMC4769843; doi:10.1186/s12864-016-2490-z)
Supplement: Additional file 2: Table S1. — Exon length comparison between SuSy BAC clone from P. pinaster and SuSy from two angiosperm plants. The first exon is lacking in the BAC clone. The gene capture model is also included (DOCX 19 kb) [file 12864_2016_2490_MOESM2_ESM.docx]

**Table S1.** Exon length comparison between *SuSy* genes from *P. pinaster* and *SuSy* from two angiosperm plants. *Non sequenced exon.

| **Exon length**  **(nt)** | **BAC *P. pinaster*** | **Gene Capture model *SuSy*** | ***Arabidopsis thaliana SuSy 2***  **(At5g49190)** | ***Arabidopsis thaliana SuSy 3***  **(At4g02280)** | ***Populus trichocarpa* (POPTRDRAFT_830445)** | ***Populus trichocarpa* (POPTRDRAFT_826368)** |
| --- | --- | --- | --- | --- | --- | --- |
| **E1** | * | 98 | 89 | 89 | 98 | 92 |
| **E2** | 133 | 133 | 133 | 133 | 133 | 127 |
| **E3** | 152 | 152 | 152 | 345 | 152 | 152 |
| **E4** | 193 | 193 | 193 |  | 193 | 193 |
| **E5** | 119 | 119 | 119 | 336 | 119 | 336 |
| **E6** | 217 | 217 | 217 |  | 217 |  |
| **E7** | 102 | 96 | 96 | 96 | 96 | 96 |
| **E8** | 174 | 174 | 174 | 291 | 174 | 174 |
| **E9** | 117 | 117 | 117 |  | 117 | 117 |
| **E10** | 167 | 167 | 167 | 167 | 167 | 167 |
| **E11** | 225 | 225 | 225 | 225 | 225 | 225 |
| **E12** | 322 | 322 | 322 | 322 | 322 | 564 |
| **E13** | 245 | 245 | 245 | 245 | 245 |  |
| **E14** | 139 | 139 | 139 | 139 | 139 | 139 |
| **E15** | 63 | 75 | 36 | 33 | 39 | 30 |
