# Supplementary material for: Establishing gene models from the Pinus pinaster genome using gene capture and BAC sequencing
Source: BMC Genomics. 2016 Feb 27;17:148. doi: 10.1186/s12864-016-2490-z (PMC4769843; doi:10.1186/s12864-016-2490-z)
Supplement: Additional file 3: Table S2. — Intron length comparisons between Susy BAC clone from P. pinaster and Susy from two angiosperm plants. The intron I8 in the BAC clone contains a gap. The gene capture model is also included (DOCX 20 kb) [file 12864_2016_2490_MOESM3_ESM.docx]

**Table S2.** Intron length comparison between *SuSy* genes from *P. pinaster* and *SuSy* from two angiosperm plants. *Non sequenced or incomplete intron.

| Intron length  (nt) | BAC *P. pinaster* | Gene Capture model *SuSy* | *Arabidopsis thaliana*  *Susy 2*  (At5g49190) | *Arabidopsis thaliana*  *Susy 3*  (At4g02280) | *Populus trichocarpa* (POPTRDRAFT_830445) | *Populus trichocarpa* (POPTRDRAFT_826368) |
| --- | --- | --- | --- | --- | --- | --- |
| I 1 | * | 208 | 110 | 88 | 95 | 127 |
| I 2 | 148 | 134 | 88 | 70 | 195 | 102 |
| I 3 | 257 | 382 | 87 | 86 | 248 | 109 |
| I 4 | 608 | 609 | 95 |  | 220 | 248 |
| I 5 | 312 | 289 | 104 | 75 | 112 | 89 |
| I 6 | 251 | 267 | 89 |  | 129 |  |
| I 7 | 32 | 268 | 103 | 97 | 105 | 108 |
| I 8 | 705* | 831 | 194 | 93 | 418 | 94 |
| I 9 | 105 | 96 | 85 |  | 95 | 98 |
| I 10 | 212 | 181 | 88 | 64 | 517 | 81 |
| I 11 | 240 | 277 | 108 | 144 | 122 | 112 |
| I 12 | 196 | 285 | 83 | 240 | 99 | 92 |
| I 13 | 262 | 149 | 88 | 82 | 75 |  |
| I 14 | 121 | 109 | 75 | 85 | 323 | 109 |
