# Supplementary material for: Establishing gene models from the Pinus pinaster genome using gene capture and BAC sequencing
Source: BMC Genomics. 2016 Feb 27;17:148. doi: 10.1186/s12864-016-2490-z (PMC4769843; doi:10.1186/s12864-016-2490-z)
Supplement: Additional file 4: Table S3. — Exon length comparison between the XET BAC clone from P. pinaster and two XET genes from Arabidopsis thaliana. The two gene capture models closest to the BAC clone are also included. (DOCX 18 kb) [file 12864_2016_2490_MOESM4_ESM.docx]

**Table S3.** Exon length comparison between *XET* genes from *P. pinaster* and two *XET* from *A. thaliana*.

| Exon length (nt) | **BAC *P. pinaster*** | **Gene Capture model**  **UniGene_27499** | **Gene Capture model**  **UniGene_23780** | ***Arabidopsis thaliana XET9* (At4g03210)** | ***Arabidopsis thaliana XET3***  **(At3g25050)** |
| --- | --- | --- | --- | --- | --- |
| E 1 | 163 | 67 | 148 | 88 | 196 |
| E 2 | 101 | 102 | 101 | 101 | 101 |
| E 3 | 194 | 194 | 194 | 194 | 194 |
| E 4 | 403 | 196 | 129 | 406 | 382 |
| E 5 | - | - | 128 | - | - |
