# Supplementary material for: Establishing gene models from the Pinus pinaster genome using gene capture and BAC sequencing
Source: BMC Genomics. 2016 Feb 27;17:148. doi: 10.1186/s12864-016-2490-z (PMC4769843; doi:10.1186/s12864-016-2490-z)
Supplement: Additional file 5: Table S4. — Intron length comparison between the XET BAC clone from P. pinaster and two XET genes from Arabidopsis thaliana. The two gene capture models closest to the BAC clone are also included. (DOCX 17 kb) [file 12864_2016_2490_MOESM5_ESM.docx]

| Intron length (nt) | **BAC *P. pinaster*** | **Gene Capture model**  **UniGene_27499** | **Gene Capture model**  **UniGene_23780** | ***Arabidopsis thaliana XET9* (At4g03210)** | ***Arabidopsis thaliana XET3***  **(At3g25050)** |
| --- | --- | --- | --- | --- | --- |
| I 1 | 123 | 215 | 112 | 119 | 88 |
| I 2 | 79 | 147 | 128 | 99 | 86 |
| I 3 | 88 | 92 | 178 | 84 | 300 |
| I 4 | - | - | 60 | - | - |

**Table S4.** Intron length comparison between *XET* genes from *P. pinaster* and two *XET* from *A. thaliana*.
