# Supplementary material for: Establishing gene models from the Pinus pinaster genome using gene capture and BAC sequencing
Source: BMC Genomics. 2016 Feb 27;17:148. doi: 10.1186/s12864-016-2490-z (PMC4769843; doi:10.1186/s12864-016-2490-z)
Supplement: Additional file 12: Table S5. — Primers used in PCR for validation of positive BAC clones in the BAC library screening. (DOCX 39 kb) [file 12864_2016_2490_MOESM12_ESM.docx]

Table S5

| Gene | cDNA Accesion number | Order number in screening filter | PCR primers | Amplified lenght (bp) |
| --- | --- | --- | --- | --- |
| Asparagine synthetase (AS) | HQ625490 | 3J16 | 5´-ACGTGCACGCCAACAATCGA-3´  5´-GCTCTGATACAGTCCACTCC-3´ | 536 |
| Xiloglucan endotransglycosilase (XET) | FN824804 | 25M3 | 5´-ACTCTGCGGGAACAGTGACT-3´  5´-CGAAGCCTTTGTAAGTGGAG-3´ | 411 |
| Sucrose synthase | AJ309093 | 26C15 | 5´-TTGGAGATCCTTCAGGCACC-3´  5´-AGGCCACACATCAAATCGAG-3´ | 841 |
