# Supplementary material for: Establishing gene models from the Pinus pinaster genome using gene capture and BAC sequencing
Source: BMC Genomics. 2016 Feb 27;17:148. doi: 10.1186/s12864-016-2490-z (PMC4769843; doi:10.1186/s12864-016-2490-z)
Supplement: Additional file 14: — Detailed description of the GeneAssembler pipeline (DOCX 124 kb) [file 12864_2016_2490_MOESM14_ESM.docx]

**GeneAssembler algorithm in detail**

The GeneAssembler pipeline was developed to build the most complex gene model that could code for each one of the 866 selected full-length proteins from the pine transcriptome. Starting with the 144,707 consensus sequences of contigs, the stages of the algorithm are: (i) gene assignment and contig filtering (Figure 2A), (ii) contig clustering by gene (Figure 2B) and (iii) gene model building (Figure 2C) to produce a gene model fro the selected full-length proteins.

*Gene assignment and contig filtering***:**

Consensus sequences of contigs were assigned to full-length proteins by BlastX search, assuming that the first match is the best (Figure 2A). Hits were filtered to discard partial similarities or spurious “high-scoring segment pairs” (hsp). Since the probes used in the gene capture were designed from cDNAs, each hsp of the BlastX result is regarded as a putative exon of the future gene model. To construct the most reliable model, assigned contigs were firstly filterer as follows: (i) the contig must present more than one hsp in an arrangement that can make think on consistent intron-exon structure (contigs having only one hsp can contain one single exon, but are discarded in this step as putative source of pseudogenes; nevertheless, they are saved in a different file for further recovery of single exon sequences in part B of the algorithm); (ii) the alignment of each hsp must not have gaps at the protein level; (iii) when a hsp is located at the 5’- or 3’-end of a gene, the sequence should be long enough to contain the entire putative exon.

Contigs that were not discarded in this filtering (“informative contigs” in Fig 2A) were compared again with the full-length proteins using EXONERATE to define more accurate intron-exon boundaries. The results were filtered as described above, resulting in reliable exon-intron structures. Additionally, frameshifts introduced by the sequencing/assembling process were resolved, reducing the amount of aberrant alignments. As a result, the informative, verified contigs (Figure 2A) have a verified intron-exon structure putatively corresponding to only one full-length protein.

*Contig clustering by gene***:**

Informative, verified contigs were clustered by full-length protein identifier (Figure 2B) to gather all contigs that can reconstruct the gene corresponding to same full-length protein. Then, single-hsp pool of contigs produced in the previous step were recovered as long as they cover an exon of the full-length that is not covered by any contig of the cluster, provided that the single-hsp, is not overlapping with previously selected contigs (since it can be considered a pseudogene). As a result, every completed gene cluster corresponding to a full-length protein contains all contigs with intron-exon, or single exon, structure that could come from the gene coding for such a full-length protein.

*Gene model building***:**

The last part of the algorithm serves to gather clusters belonging to the same gene to obtain the corresponding model (Figure 2C). Contigs are then sorted along the putative gene using two different criteria:

1. a blind alignment of contigs with respect to themselves, in a position-based approach, as an attempt to locate every exon based only in its coordinates of the alignment; and
2. a guided alignment of exons with the putative ortholog of this this full-length in four model plants obtained from Phytozome.

The obtained alignments are then represented in a bidimensional Boolean array of contigs vs exons, where 1 means that the exon is covered by a contig, and 0 indicates that the exon was not captured.

The set of sorted contigs is then simplified when a contig contained in longer contigs is detected on the basis of the exons shared by both contigs (Figure 2C, “removing redundant contig” box). The optimal reduction would provide a single contig covering all exons, but more commonly several contigs are required to cover the whole genomic sequence of the gene.

Then, the minimal number of contigs covering a gene are merged in a single sequence to generate the corresponding gene model, discarding exons in overlapping boundaries and concatenating non-overlapping contigs (signaling the gaps when the contigs are not correlative) (Figure 2C, “Contig mergig” box). This results in one gene model for the blind alignment, and at least one gene model for every one of the four guiding plants used.

The best gene model is the one that comply with the following criteria: (i) the one recovering more sequence of the seeding full-length, (ii) the one with the lowest overlap percentage of contigs at the exon level; and (iii) the one with the lowest fragmentation index, that is, the one obtained from the fewest number of contigs.

The GeneAssembler gene model for each full-length is saved in a gff3 file including the contigs that belong to it, a FASTA file with the gene model sequences, and an index that relates each contig with its full-length.
